# Supplementary material for: Contextual factors associated with contraceptive utilization and unmet need among sexually active unmarried women in Kenya: A multilevel regression analysis
Source: PLoS One. 2022 Jun 30;17(6):e0270516. doi: 10.1371/journal.pone.0270516 (PMC9246151; doi:10.1371/journal.pone.0270516)
Supplement: S1 Appendix — (DOCX) [file pone.0270516.s001.docx]

**Title**

Contraceptive Demand and Utilization by Unmarried, Sexually Active Women in Kenya: A Multilevel Regression Analysis

**Appendix**

**Table A.1: Indicator Definitions**

|  | Exploratory Variable | Definition | Categories | Questionnaire |
| --- | --- | --- | --- | --- |
|  | Socio-demographic, Geographic and Economic |  |  |  |
| 1 | Age Group | Age of the female respondent (by category) | 15-24 (ref); 25-34; 35-44; 45-49 | Female |
| 2 | Parity | Number of previous children for the female respondent (by category) | None (ref); 1-2; 3-4; 5 plus | Female |
| 3 | Education | Highest level of schooling attended by the female respondent | Never (ref); Primary; Secondary or more | Female |
| 4 | Household wealth quintile | Wealth of the household calculated by PMA using a principal components analysis of household construction and assets (categorized by quintile) | Lowest (ref); Middle lowest; Middle; Middle highest; Highest | Household |
| 5 | Residence | Location of residence (categorized by PMA as Urban or Rural) | Urban (ref); Rural | Household |
| 6 | Female subgroups | Female respondent's marital status including subclassification of unmarried women by recency of sexually activity | Unmarried sexually active (1-12 months) (ref); Unmarried sexually active (0-30 days); Married or in union | Female |
|  | Service Delivery Point |  |  |  |
| 7 | Number of methods offered at SDPs | Within the EA, average number of methods provided to clients across facilities | 0-2 methods (ref); 3-4 methods; 5 or more methods | Service Delivery Point (SDP) |
| 8 | Number of methods offered at NO charge at SDPs | Within the EA, average number of methods provided to clients that do NOT change a fee across facilities | 0-2 methods (ref); 3-4 methods; 5 or more methods | Service Delivery Point (SDP) |
| 9 | Fees for health provider | Within the EA, does any facility NOT charge family planning clients to see a health provider | At least one (1) facility in the EA does NOT charge a fee (ref); All facilities in EA charge a fee | Service Delivery Point (SDP) |
| 10 | Stockout of any method offered in the last 3 months | Within the EA, has any facility had a stockout in the last 3 months of any method that is offered | No (ref); Yes, at least one (1) facility in the EA had a stockout in the last 3 months | Service Delivery Point (SDP) |
| 11 | Stockout of any short-acting methods offered | Within the EA, has any facility had a stockout in the last 3 months of any short-acting method that is offered | No (ref); Yes, at least one (1) facility in the EA had a stockout in the last 3 months | Service Delivery Point (SDP) |
| 12 | Stockout of any long-acting methods offered | Within the EA, has any facility had a stockout in the last 3 months of any long-acting method (except male and female sterilization) that is offered | No (ref); Yes, at least one (1) facility in the EA had a stockout in the last 3 months | Service Delivery Point (SDP) |
|  | Demand, Contact and Satisfaction |  |  |  |
| 13 | Visited by health worker who talked about family planning in last 12 months | Female respondent was visited by health worker who talked about family planning in last 12 months | No (ref); Yes | Female |
| 14 | Heard family planning message in the last few months | Female respondent had heard family planning message in the last few months | No (ref); Yes | Female |
| 15 | Visited a health facility in last 12 months | Female respondent visited a health facility in last 12 months | No (ref); Yes | Female |
| 16 | Visited a health facility in last 12 months and consulted on family planning | Female respodent visited a health facility in last 12 months and consulted on family planning | No (ref); Yes | Female |
| 17 | Female respondent would return to this provider | Female respondent reported that she would return to this provider | No (ref); Yes | Female |
| 18 | Female respondent would refer her relative or friend to this provider / facility | Female respondent reported that she would refer her relative or friend to this provider / facility | No (ref); Yes | Female |
| 19 | Female respondent would return to this provider OR refer her relative or friend to this provider / facility | Female respondent reported that she would return to this provider OR refer her relative or friend to this provider / facility | No (ref); Yes | Female |

Note: Indicators #7, 8, 10, 11, 12, 13, 15, 16, 17, 18, and 19 were evaluated, but ultimately not included in the final models.

**Table A.2: Contraceptive Indicators by Female Subgroups**

| **Modern CPR (mCPR)** |  | **2014** | **2017** | **2019** | **Change 2014-19** |
| --- | --- | --- | --- | --- | --- |
|  | Unmarried Sexually Active (1-12 months) | 35.0% | 39.3% | 44.1% | 9.1% |
|  | Unmarried Sexually Active (0-30 days) | 46.1% | 57.6% | 62.1% | 16.0% |
|  | Married or in union | 53.4% | 60.0% | 58.8% | 5.4% |
|  |  |  |  |  |  |
| **Unmet Need** |  | **2014** | **2017** | **2019** | **Change 2014-19** |
|  | Unmarried Sexually Active (1-12 months) | 6.1% | 13.2% | 13.5% | 7.4% |
|  | Unmarried Sexually Active (0-30 days) | 47.0% | 31.4% | 22.6% | -24.4% |
|  | Married or in union | 24.8% | 15.3% | 14.8% | -10.0% |
|  |  |  |  |  |  |
| **Total Demand for Modern Contraceptives** |  | **2014** | **2017** | **2019** | **Change 2014-19** |
|  | Unmarried Sexually Active (1-12 months) | 41.1% | 52.6% | 57.6% | 16.5% |
|  | Unmarried Sexually Active (0-30 days) | 93.1% | 89.0% | 84.7% | -8.4% |
|  | Married or in union | 78.3% | 75.3% | 73.6% | -4.7% |
|  |  |  |  |  |  |
| **% Demand Satisfied by Modern Contraceptives** |  | **2014** | **2017** | **2019** | **Change 2014-19** |
|  | Unmarried Sexually Active (1-12 months) | 85.2% | 74.8% | 76.6% | -8.6% |
|  | Unmarried Sexually Active (0-30 days) | 49.5% | 64.7% | 73.3% | 23.8% |
|  | Married or in union | 68.3% | 79.7% | 79.9% | 11.6% |
|  |  |  |  |  |  |
| **Recent Emergency Contraceptive** |  | **2014** | **2017** | **2019** | **Change 2017-19** |
|  | Unmarried Sexually Active (1-12 months) | na | 10.6% | 13.5% | 2.9% |
|  | Unmarried Sexually Active (0-30 days) | na | 14.1% | 13.3% | -0.8% |
|  | Married or in union | na | 5.4% | 7.2% | 1.8% |
|  |  |  |  |  |  |
| **Unmet Need - For Spacing** |  | **2014** | **2017** | **2019** | **Change 2014-19** |
|  | Unmarried Sexually Active (1-12 months) | 4.7% | 8.8% | 9.2% | 4.5% |
|  | Unmarried Sexually Active (0-30 days) | 32.1% | 26.2% | 15.2% | -16.9% |
|  | Married or in union | 12.9% | 8.3% | 8.1% | -4.8% |
|  |  |  |  |  |  |
| **Unmet Need - For Limiting** |  | **2014** | **2017** | **2019** | **Change 2014-19** |
|  | Unmarried Sexually Active (1-12 months) | 1.3% | 4.4% | 4.2% | 2.8% |
|  | Unmarried Sexually Active (0-30 days) | 14.9% | 5.2% | 7.4% | -7.4% |
|  | Married or in union | 11.9% | 7.0% | 6.7% | -5.2% |
